# Identification of AKNA Gene and Its Role for Genetic Susceptibility in Epithelial Ovarian Cancer

**DOI:** 10.3390/cimb47020078

**Published:** 2025-01-26

**Authors:** Dwi Anita Suryandari, Miftahuzzakiyah Miftahuzzakiyah, Luluk Yunaini, Ria Kodariah, Dewi Sukmawati, Primariadewi Rustamadji, Puji Sari, Sri Suciati Ningsih

**Affiliations:** 1 Department of Medical Biology, Faculty of Medicine, Universitas Indonesia, Jakarta 10430, Indonesia; lulukyunaini@ui.ac.id (L.Y.); pudji.sari@ui.ac.id (P.S.); 2 Biobank Research IMERI, Faculty of Medicine, Universitas Indonesia, Jakarta 10430, Indonesia; ria.kodariah@ui.ac.id; 3 Master Program in Biomedical Biology, Faculty of Medicine, Universitas Indonesia, Jakarta 10430, Indonesia; zakiyahsyarifuddin@gmail.com; 4 Department of Anatomical Pathology, Faculty of Medicine, Universitas Indonesia, Jakarta 10430, Indonesia; primariadewi.rustamadji@ui.ac.id; 5 Department of Histology, Faculty of Medicine, Universitas Indonesia, Jakarta 10430, Indonesia; ds_histoui@outlook.com; 6 Doctoral Program in Biomedical Sciences, Faculty of Medicine, Universitas Indonesia, Jakarta 10430, Indonesia; sri.suciati11@ui.ac.id

**Keywords:** AKNA, genetic susceptibility, epithelial ovarian cancer

## Abstract

AKNA is identified as a gene that regulates inflammation, immune response, and Epithelial–Mesenchymal Transition (EMT), which plays an important role in the progression of epithelial ovarian cancer. In this study, we analyzed the genotype and allele distribution as well as 3D modeling of one of the AKNA rs10817595 (−1372 C>A). The distribution of genotypes and alleles was analyzed using the T-ARMS PCR method on 63 ovarian cancer samples and 65 controls. AKNA mRNA expression was analyzed using qRT-PCR on 35 low-grade and 28 high-grade samples. Fifteen low-grade and 12 high-grade samples were analyzed for AKNA protein levels using immunohistochemistry. A 3D model of protein structure was constructed using AlphaFold. Significant differences in AKNA protein levels were found. However, no significant correlation was found for relative AKNA mRNA expression with protein levels. This result is thought to be related to decreased immune system response, increased inflammation, and increased EMT in epithelial ovarian cancer. AKNA gene variant (−1372 C>A) can cause a decrease in mRNA and protein levels in the low-grade and high-grade groups, so it has the potential as a genetic susceptibility factor in epithelial ovarian cancer.

## 1. Introduction

Ovarian cancer, a gynecological disease, is known as the “silent killer” due to the high mortality rate and the lack of early screening methods resulting in delays in diagnosis [1]. There are several risk factors that can increase the incidence of ovarian cancer. One of them is genetic susceptibility factors to ovarian cancer [2]. Based on the Dualistic Model, epithelial-type ovarian cancer is classified into low-grade and high-grade types. The low-grade type represents early-stage ovarian cancer, low proliferative activity, rare ascites, generally good prognosis, and a high risk of developing endometriosis. The high-grade type is usually characterized by the presence of TP53 mutations, most of which are advanced ovarian cancers, high proliferation, BRCA mutations occur, poor prognosis, and usually ascites [3]. One of the causes of ovarian carcinogenesis is repeated ovulation events that trigger the transformation of epithelial cells [4]. At the initiation stage of tumorigenesis, the biological response of the body’s immune system is also involved in eliminating cancer antigens [5]. One of the genes that has a role in regulating inflammation, EMT, and anti-tumor immune response is the AKNA gene [6].

AKNA is encoded by a single gene located in the FRA9E region of chromosome 9q32, which is known to have the potential for mutations that cause inflammation and cancer [7]. AKNA is widely expressed in the germinal center of secondary lymphoid organs and ovaries [8,9]. AKNA encodes the AT-Hook Transcriptional Factor, a core protein that can influence the process of carcinogenesis [10]. AKNA transcription factor can enhance anti-tumor immunity by interacting with CD154 ligands and CD40 receptors [11]. The AKNA transcription factor also plays a role in repressing the expression of the inflammatory genes MMP-9, interferon 1β, and neutrophil granule protein. Low AKNA expression increases the secretion of proinflammatory cytokines, thereby triggering chronic inflammation and the transition of cancer to become more malignant [12]. The protein E6/p53/AKNA complex is thought to play an essential role in the immune system’s deregulation during cervical carcinogenesis [13]. In GH3 cells, AKNA expression decreased significantly after exposure to T-2 toxin; it is suspected that AKNA is an essential regulator of T-2 toxin by inducing inflammatory cytokine [14].

Initially, the role of AKNA was known in eliminating cancer antigens through an inflammatory response leading to an increase in chronic inflammation for cancer [15]. The AKNA transcription factor plays a significant role in the immune response and inflammation associated with ovarian cancer. Research indicates that AKNA expression varies across different tumor grades, suggesting its potential as a predictive biomarker. This overview will explore AKNA’s expression patterns, its relationship with immune mediators, and the implications for ovarian cancer progression. The differential expression of AKNA may correlate with the tumor microenvironment’s inflammatory profile, influencing immune cell recruitment and activity [16]. Previous studies have proven that one variant of the AKNA promoter, namely rs10817595 (−1372 C>A), is associated with Squamous Intraepithelial Lesion (SIL) and cervical cancer [8]. AKNA variants in the promoter can affect the expression of AKNA, which is thought to have implications for the regulation in modulating the expression of genes related to inflammation, carcinogenesis, and cancer progression [17]. The interplay between AKNA expression and inflammatory markers may provide insights into patient stratification and therapeutic approaches, as higher inflammatory responses are often associated with worse prognosis [18].

The aim of this study is to analyze AKNA gene variant (−1372 C>A) distribution and expression in normal and ovarian cancer in the Indonesian population. This study also analyzed differences in the expression of AKNA mRNA and protein levels in the low-grade and high-grade groups and the correlation of AKNA mRNA expression with AKNA protein expression level. A 3D model is employed to detect whether the point mutation affects the alteration of the amino acid sequence of the AKNA protein. This study is expected to serve comprehensive insights about the role of AKNA in pathogenesis and as a predictor of genetic susceptibility to epithelial ovarian cancer.

## 2. Materials and Methods

### 2.1. Study Design and Sample Collection

A cross-sectional design was conducted in this research. The approval research was obtained from the University of Indonesia Medical Research Ethics Committee (Number Ket-617/UN2.F1/ETIK/PPM.00.02/2020). In ovarian cancer, as many as 65 Formalin-Fixed Paraffin-Embedded (FFPE) tissue samples were used for genotype and allele analysis. The control group in genotype analysis was peripheral blood from blood donor patients (non-cancer) obtained from the Indonesian Red Cross Central Jakarta.

### 2.2. Genotyping of AKNA (−1372 C>A) Using Tetra-Primer Amplification Refractory Mutation System-Polymerase Chain Reaction (T-ARMS PCR)

Ovarian cancer FFPE tissue was microdissected, then deparaffinized, and DNA was isolated using the gSYNCTM DNA Extraction Kit (GS100, New Taipei City, Taiwan). While the isolation of DNA from blood as a control sample was carried out using the salting out method. The primers used for the T-ARMS reaction were designed using primer1soton software with amplicon length criteria ranging from 80 bp to 160 bp. The length of the amplicon considered from the sample used is in the form of tissue in a paraffin block. T-ARMS PCR requires four primers. A pair of Forward Outer primers 5′- CTC TCA CAG AAC CCC ATA A -3′ and a Reverse Outer primer 5′- TGA CAC ATG GTA GGG ATT C -3′ as internal controls produced 150 bp amplicon; a pair of Forward Outer primers and Reverse Inner primers 5′- GTC TAC CTT CAC AGA GTT TTT TT -3′ will recognize specific A alleles to produce 101 bp amplicons; a pair of Forward Inner primers 5′- TGT AAC CAG TTC AAC TCC AC -3′ and Reverse Outer primers recognize specific C alleles with length amplicons of 91 bp. PCR reactions were then carried out at annealing temperature of 510 °C for all primers. Followed by electrophoresis on 3% agarose gel. Each sample consists of 3 wells on the electrophoretic gel. Internal control showed 150 bp, C allele 91 bp, and A allele 101 bp. The CC homozygous genotype is marked with 2 bands: internal control band (150bp) and C allele (91 bp). CA heterozygous genotype is characterized by 3 bands: control internal band (150 bp), C allele band (91 bp), and A allele band (101 bp). The AA homozygous genotype is indicated by 2 bands: internal control band (150 bp) and allele A band (101 bp).

### 2.3. mRNA Relative Expression of AKNA Using Quantitative Reverse Transcriptase Real-Time Polymerase Chain Reaction (qRT-PCR)

This study used two-step qRT-PCR. Quick-RNA Miniprep Plus Kit from Zymo (R1057, Tustin, CA, USA) was used for RNA isolation, and ReverTra AceTM qPCR RT Master Mix with gDNA Remover Toyobo (FSQ-101, Japan) was used for cDNA synthesis. AKNA gene primers, namely Forward primer 5′-GAG GTG AAG AGC AGA TTG TC -3′ and Reverse primer 5′- GAT TCT GAA CTC AGG GAC AG -3′. Normalization using the GAPDH gene with primer Forward 5′- GAA ATC CCA TCA CCA TCT TCC AGG -3′ and primer Reverse 5′-GAG CCC CAG CCT TCT CCA TG -3′. The qRT-PCR reaction uses an annealing temperature of 600 °C. The value of the threshold cycle (Ct) obtained from qRT-PCR of the AKNA gene was processed using the Livak method [19].

### 2.4. AKNA Protein Analysis Using Immunohistochemistry

The immunohistochemistry (IHC) method was performed to assess AKNA protein distribution and expression in ovarian tissue. Tonsil tissue served as a positive control, using a primary antibody dilution of 1:100. FFPE ovarian tissue sections were dried, deparaffinized, rehydrated, and blocked with 3% hydrogen peroxide. Slides were incubated overnight with a primary anti-AKNA antibody (Fine-Test, Wuhan, China) and subsequently treated with a secondary antibody, streptavidin-HRP, and DAB substrate. Haematoxylin counterstaining, dehydration, clearing, and mounting were then performed. Microscopic analysis at 400× magnification evaluated five fields of view per sample, with staining quantified using ImageJ Fiji. Staining intensity was scored using a modified H-score method based on the percentage of cells with weak (1), moderate (2), or strong (3) staining. Scores were interpreted as follows: <50 (negative), 50–100 (weakly positive, 1+), 101–200 (moderately positive, 2+), and 201–300 (strongly positive, 3+). Results were verified by the researcher and an academic advisor to ensure accuracy [20].

### 2.5. Statistical Data Analysis

The statistical analysis used was SPSS 22. Differences in the distribution of AKNA genotypes and alleles of SNP in ovarian cancer and the control group were tested with a chi-square test. Differences in AKNA protein and mRNA expression in low-grade, high-grade, and cyst epithelial ovarian cancer using the Kruskal–Wallis test, followed by a post-hoc test. Correlation of AKNA mRNA with AKNA protein expression in low-grade and high-grade ovarian cancer tested with the Spearman correlation test.

## 3. Results

### 3.1. Genotyping of AKNA (−1372 C>A) in Epithelial Ovarian Cancer

**Figure 1:**
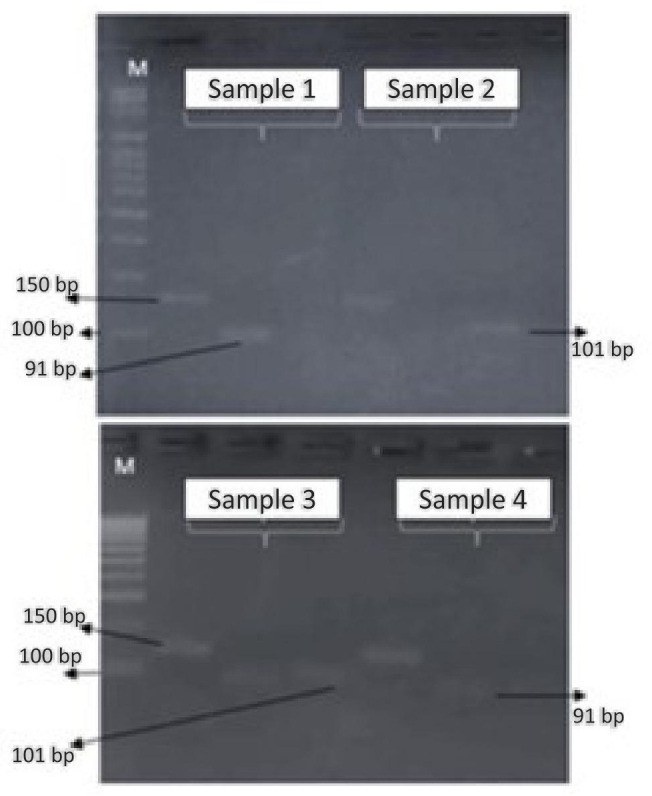
Results of electrophoretic visualization. Sample 1: CC homozygous, sample 2: AA homozygous, sample 3: CA heterozygous, and sample 4: CC homozygous. M: markers 100 bp.

Visualization results of AKNA rs10817595 (C>A) gel electrophoresis in the ovarian cancer group and controls using T-ARMS PCR can be seen in Figure 1. The frequency distribution of the A allele in the ovarian cancer group was higher than that of the C allele. The same was true for the control group. The statistical results of the genotype and allele frequencies distribution showed no significant difference in the ovarian cancer group and the control group with a *p*-value> 0.05 (Table 1). However, statistical tests on combined genotypes showed that the AA homozygous genotype had a risk factor (OR = 0.026, 95% CI 2.687) for CA and CC genotypes in epithelial ovarian cancer (Table 2).

### 3.2. Relative Expression of AKNA mRNA in Low-Grade and High-Grade Epithelial Ovarian Cancer Compared with Ovarian Cysts

**Figure 2:**
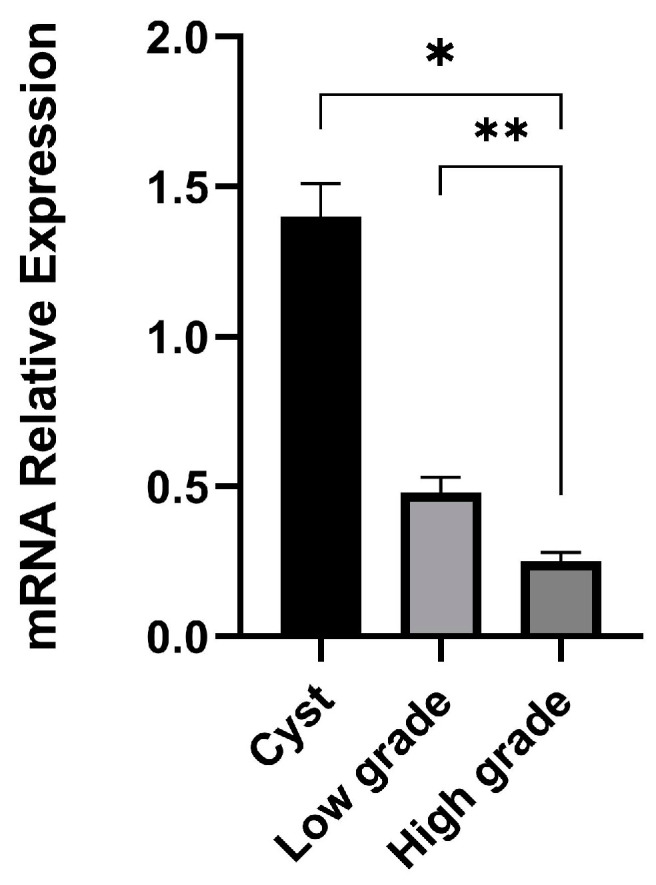
mRNA relative expression of AKNA mRNA (Kruskal–Wallis, * *p* < 0.05; ** *p* < 0.01).

The results showed a statistically significant difference in the relative expression of AKNA in ovarian cancer and cyst groups (*p* = 0.002) (Figure 2). The relative expression of the AKNA in the low-grade ovarian cancer group decreased 1.42 times that of the cyst group. The expression in the high-grade group was 3.67 times lower than the cyst group. Post-hoc tests using the Mann–Whitney showed that there were significant differences in the “high-grade and cyst” group and the “low-grade and high-grade” group.

### 3.3. Figures, Tables, and Schemes

**Figure 3:**
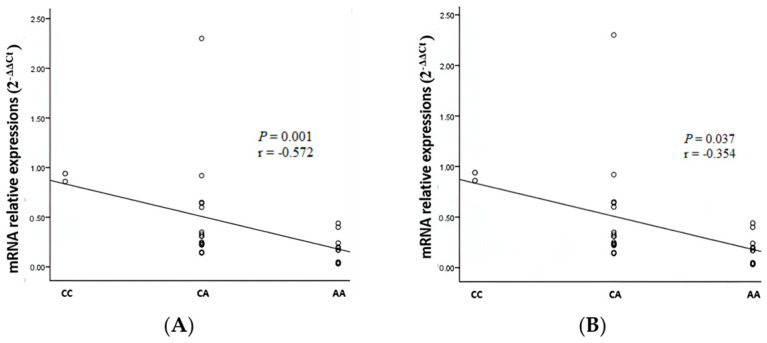
Graph of AKNA genotype correlation (−1372 C>A) with relative expression of AKNA mRNA (−1372 C>A) in the low-grade group (**A**) and high-grade group (**B**).

A significant negative correlation was found between the AKNA genotype rs10817595 (−1375 C>A) and the relative expression of AKNA mRNA in low-grade epithelial ovarian cancer (*p* = 0.037, r = −0.354) (Figure 3A) and high-grade epithelial ovarian cancer (*p* = 0.001, r = −0.572) (Figure 3B). The linearity graph shows a negative correlation, meaning that the AA genotype has a lower relative expression of AKNA mRNA than the CA and CC genotypes.

### 3.4. AKNA Protein Expression in Low-Grade and High-Grade Ovarian Epithelial Cancer Compared with Ovarian Cysts

**Figure 4:**
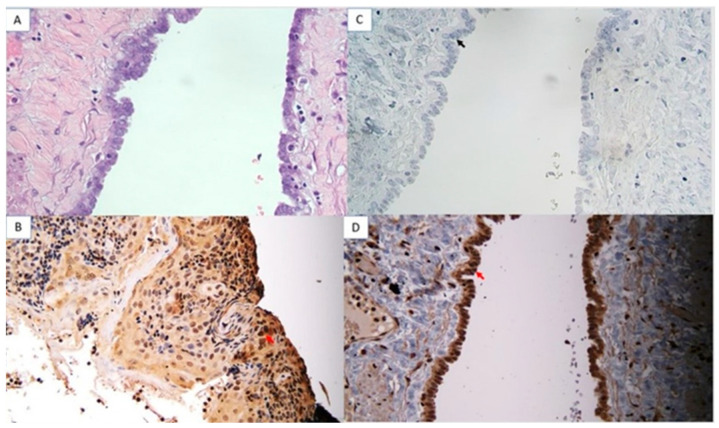
AKNA protein expression using immunohistochemistry in ovarian cyst (40× magnification). (**A**) Hematoxylin Eosin (HE) ovarian cyst. (**B**) Positive control (tonsil tissue with dark brown–black epithelial cell nuclei (red arrows)). (**C**) Negative control (ovarian cysts with blue nuclei of epithelial cells (black arrows)). (**D**) Ovarian cyst tissue (positive AKNA protein with dark brown–black color (red arrow).

**Figure 5:**
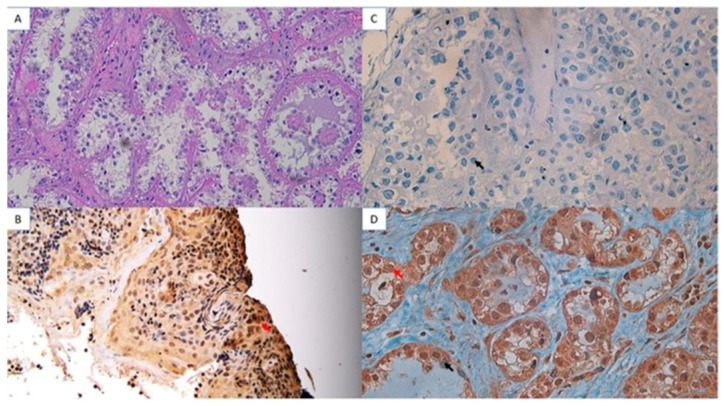
AKNA protein expression using immunohistochemistry in low-grade ovarian epithelial cancer (40× magnification). (**A**) HE, (**B**) Positive control (tonsil tissue with dark brown–black epithelial cell nuclei (red arrows)). (**C**) Negative control (ovarian cysts with blue nuclei of epithelial cells (black arrows)). (**D**) Ovarian cyst tissue (positive AKNA protein with dark brown–black color (red arrow).

**Figure 6:**
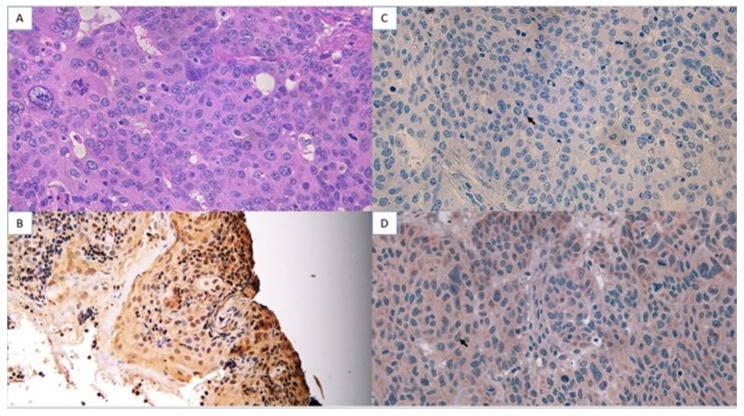
AKNA protein expression using immunohistochemistry in high-grade ovarian epithelial cancer (40× magnification). (**A**) HE, (**B**) Positive control (tonsillar tissue), (**C**) Negative control, (**D**) High-grade ovarian cancer tissue treated with anti-AKNA antibody. AKNA protein in the cell nucleus, marked in blue (black arrow).

**Figure 7:**
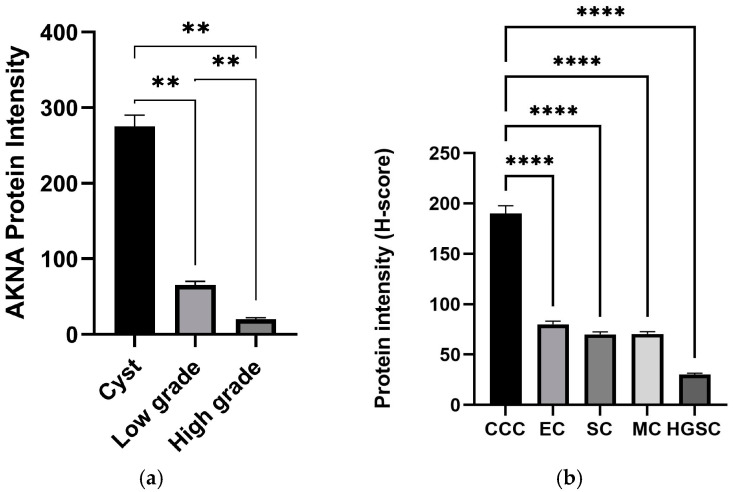
AKNA protein expression (**a**) and the intensity of AKNA protein expression (**b**). (Kruskal–Wallis, Mann–Whitney, ** *p* < 0.01; **** *p* < 0.0001). CCC: Clear Cell Carcinoma (*n* = 4), EC: Endometroid Carcinoma (*n* = 3), SC: Serous Carcinoma (*n* = 1), MC: Mucinous Carcinoma (*n* = 7), high-grade consists of HGSC: High-grade Serous Carcinoma (*n* = 12).

The analysis of AKNA protein expression using immunohistochemistry showed a strong intensity of AKNA protein expression in cysts (Figure 4) and decreased intensity in low-grade ovarian cancer (Figure 5) and high-grade (Figure 6). The AKNA protein expression in the low-grade and high-grade epithelial ovarian cancer groups was lower than that of cysts (Figure 7a). Based on this grouping, AKNA protein expression in CCC was higher than in other low-grade epithelial ovarian cancers (Figure 7b).

### 3.5. Correlation Between AKNA mRNA Expression and AKNA Protein Expression in Low-Grade and High-Grade Epithelial Ovarian Cancer

**Figure 8:**
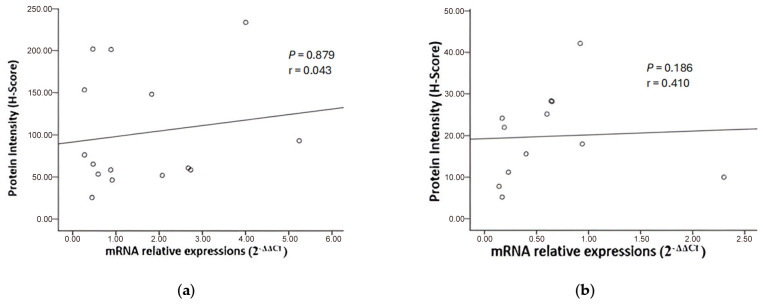
Relative correlation of AKNA mRNA expression with AKNA protein expression in the low-grade group (**a**) and in the high-grade group (**b**).

Spearman correlation test results of AKNA mRNA relative expression with AKNA protein expression in low-grade ovarian cancer showed no significant correlation (*p* = 0.879, r = 0.043) (Figure 8a). It was similar in high-grade ovarian cancer (*p* =0.186, r = 0.410) (Figure 8b).

## 4. Discussion

This study found that the AA genotype had a risk factor for ovarian cancer compared with the CA and CC genotypes. Therefore, individuals with the AA genotype are more likely to have a risk of epithelial ovarian cancer based on the combined genotype analysis. This result is in line with Hernandez-Molina et al. that the AKNA variant (−1372 C>A) can increase the risk of primary Sjogren’s syndrome (pSS) disease by 2.60 times (95% CI) [21]. In addition, the AKNA variant (−1372 C>A) is also a genetic susceptibility factor for cervical cancer in the Mexican population and knee osteoarthritis (KOA) in the Chinese and Mexican populations [22,23]. The AKNA gene is reported to be located in the FRA9E region associated with inflammation and ovarian carcinogenesis [24]. AKNA (−1372 C>A) is a polymorphic variant; however, the AKNA genotype and allotype analysis in this study were not significantly different in the Indonesian population, but the combined genotype results showed that the AA genotype has a risk factor for the CC and CA genotypes in epithelial ovarian cancer. Therefore, a larger sample and further genotypic studies are needed to determine the association of the AKNA (−1372 C>A) variant with ovarian cancer in the population of Indonesia.

This study showed a statistically significant difference between the high-grade and cyst groups and the low-grade as well. This difference might be due to the deregulation of transcription of the AKNA in the high-grade group [4,13]. The correlation test in the low-grade and high-grade groups showed a statistically significant correlation between the AKNA rs10817595 genotypes. These results align with previous results showing that the AA genotype AKNA variant rs10817595 poses a higher ovarian cancer risk compared with the CA and CC genotypes. This variant in the promoter region likely impacts AKNA transcription, as suggested by its lower expression in the AA genotype within the Indonesian population. These results were supported by the study of Zhao et al., which stated that AKNA rs10817595 could cause a decrease in AKNA expression levels in the Chinese population [22]. The reduced AKNA mRNA expression in the AA genotype may heighten inflammation, leading to excessive tissue repair during ovulation and a pro-carcinogenic tumor microenvironment that increases epithelial ovarian cancer risk. AKNA regulates immune response gene expression and dampens inflammation, so loss of AKNA function may impair immune defense and facilitate tumor growth [8].

Immunohistochemistry showed reduced AKNA protein expression in both low- and high-grade epithelial ovarian cancer, with lower intensity in low-grade cancer than in cysts and even lower in high-grade cancer (Figure 4, Figure 5 and Figure 6). Statistical analysis confirmed significant differences in AKNA protein levels across cyst, low-grade, and high-grade groups. The decrease in AKNA protein expression is thought to be due to AKNA being activated to eliminate cancer pathogens, leading to increased inflammation in the cancer microenvironment, EMT imbalance, elevated MMP-9, and enhanced cancer colonization. This result aligns with Wang et al.’s findings, which reported decreased AKNA mRNA expression in gastric cancer cell lines compared with normal gastric epithelial cells (GES-1 cell lines). The results of the Gene Set Enrichment Analysis (GSEA) explained that AKNA could regulate cell-to-cell adhesion, T-cell proliferation, chemokine signaling pathways, the interaction of cytokine receptors with cytokines, cell adhesion molecule interactions, and JAK-STAT signaling pathways that are related to EMT [17]. We propose that AKNA may inhibit uncontrolled EMT in epithelial ovarian cancer through the expression of E-cadherin, a tumor-suppressor adhesion molecule. Low E-cadherin in ovarian cancer correlates with poor prognosis, and in gastric cancer, low AKNA expression is also associated with reduced E-cadherin [25,26]. Given AKNA’s role as a centrosomal protein regulating microtubule stability, its low expression in ovarian cancer may lead to decreased E-cadherin and metastasis via uncontrolled EMT [15].

Despite the observed decrease in AKNA mRNA and protein expression, Spearman correlation analysis showed no significant link between their levels in low- and high-grade epithelial ovarian cancer. Previous studies also report weak or insignificant mRNA-protein correlations. For example, MMP-9 analysis in prostate cancer showed elevated protein levels in cancer tissue, but without a corresponding mRNA-protein correlation. Andrieux et al. suggested that tumor microenvironments can disrupt mRNA-protein relationships, while Liu et al. argued that mRNA levels alone are insufficient for predicting protein levels, emphasizing the need for large, high-quality datasets [27,28]. Additionally, Schwanhausser et al. noted that genes related to transcription factors and cell cycles often have unstable mRNA and protein, leading to weak correlations [29]. Mechanisms like polyadenylation and poor folding of AKNA’s PEST region further disrupt mRNA-protein expression correlation [24]. Furthermore, in Figure 7b, we grouped low-grade types (endometrioid, mucinous, serous, and CCC) and found that AKNA protein expression was higher in CCC than in other types. This may be due to CCC’s low TP53 mutation rate (<10%) and the abundance of clear cytoplasmic fluid, which may aid AKNA translocation to the nucleus to regulate EMT-related pathways, preventing malignancy. However, the difference in AKNA expression across low-grade types may also stem from sample size variations. AKNA contains multiple PEST protein cleavage motifs that directly bind to A/T-rich regulatory elements of the promoter region of CD40 and CD40 that played an important role in carcinogenesis through the deregulation of the immune system in cancer [18,30].

**Figure 9:**
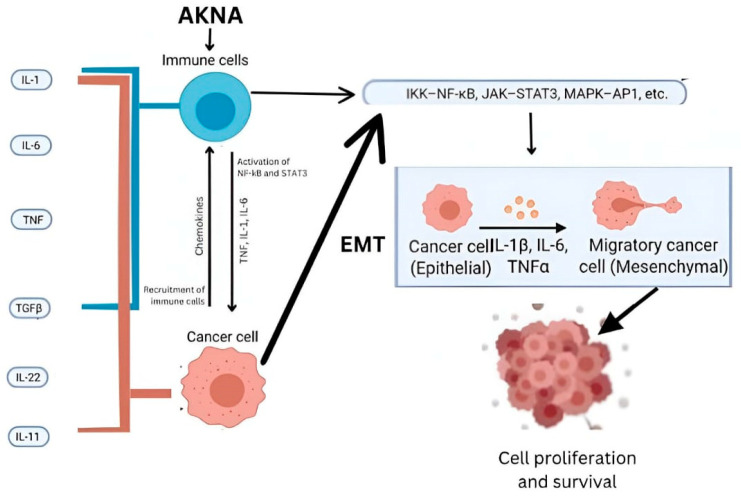
Pathway involving the AKNA gene and its influence on immune cells and cancer cells.

Figure 9 proposes an illustration pathway involving the AKNA gene and its influence on immune cells and cancer cells, particularly in the context of cancer progression and metastasis. The AKNA gene influences immune cells, promoting the activation of transcription factors like NF-κB and STAT3. This activation leads to the production of chemokines and cytokines (e.g., TNF, IL-1, IL-6), potentially enhancing the inflammatory response. Cytokines such as IL-1, IL-6, TNF, and others (TGF-β, IL-22, IL-11) interact with cancer cells, promoting signaling pathways (e.g., IKK-NF-κB, JAK-STAT3, MAPK-AP1) that support cancer progression. This signaling can initiate the EMT process, which contributes to metastasis. EMT with epithelial cancer cells (shown as rounder shapes) transforming into mesenchymal cancer cells (elongated shapes). EMT allows cancer cells to become more migratory and invasive.

## 5. Conclusions

In conclusion, the AA genotype of the AKNA variant (−1372 C>A) can cause a decrease in AKNA mRNA expression and AKNA protein expression in low-grade and high-grade ovarian cancer patients. This result is thought to be related to decreased immune system response, increased inflammation, and increased EMT in epithelial ovarian cancer. Thus, the AA genotype has the potential as a genetic susceptibility factor in low-grade and high-grade epithelial ovarian cancer.

## Figures and Tables

**Table 1:** Frequency distribution of *AKNA* genotypes and alleles (−1372 C>A) and results of the chi-square test statistic (α = 0.05) in epithelial ovarian cancer.

*AKNA* (−1372 C>A)			
Genotype	Frequency		*p*-Value
	Cancer (*n* = 63)	Control (*n* = 65)	
CC	0.095	0.123	0.08
CA	0.603	0.738	
AA	0.301	0.138	
Allele	Cancer (*n* = 126)	Control (*n* = 130)	0.12
C	0.397	0.492	
A	0.603	0.508	

**Table 2:** Combined genotype frequency of *AKNA* in epithelial ovarian cancer.

Genotype	Frequency of Genotype				*p*-Value	OR (CI 95%)
	Control		Cancer			
	I^CC^	II^CA+AA^	I^AA^	II^CA+AA^		
CC vs. (CA and AA)	0.123	0.876	0.095	0.904	0.61	0.75 (0.24–2.30)
AA vs. (CA and CC	0.138	0.861	0.301	0.698	0.02	2.68 (1.11–6.51)

## Data Availability

All data are available in this manuscript.
